# Continuous suture technique in aortic valve replacement

**DOI:** 10.1186/1749-8090-8-S1-O31

**Published:** 2013-09-11

**Authors:** I Panagiotopoulos, I Linardakis, K Katsavrias, D Pavlopoulos, S Prapas

**Affiliations:** 1 Cardiac Surgery Dpt., Henry Dunant Hospital, Athens, Greece

## Background

The aim of the study is to describe our experience in using continuous suture technique for aortic valve replacement and to evaluate the possible advantages of it.

## Methods

Between February 2001 and October 2011, a total number of 443 patients with aortic valve stenosis underwent aortic valve replacement. It was isolated in 217 patients (group A), combined with coronary vascularisation in 137 cases (group B) and with other concomitant cardiac procedures in 89 cases (group C). In all groups continuous suture technique was used. The most of coronary anastomosis performed “off pump” and only few of them on a beating heart with the support of the pump. Valves used were metallic in 247 cases, stented in bioprostheses in 188 cases and unstented bioprostheses in 8 cases. Sizes of the valves varied from 19 to 27. The valve had always the size of the annulus except for few cases in which the annulus was greater than 27.

## Results

The cross-clamp time and bypass time were similar in groups A and B and significantly longer in group C. No perivalvural leak was detected in entry postoperative echocardiograms. There was no need for aortic annulus enlargement. Hospital mortality was 3,5 % in group A, 4,3 % in group B and 3,3 % in group C, respectively. There were no deaths or complications associated with suture technique. During the follow-up period 2 patients of group A and 1 patient of group C developed perivalvural leakage requiring reoperation. The reason was tearing of the annulus tissue. In all cases the metallic valves placed were smaller in size than the enlarged annulus size.

## Conclusions

Continuous suture method is safe and useful for aortic valve replacement technique. It is simple, quick and effective, saving bypass and cross clamp time in benefit of other cardiac procedures. Combined with off pump coronary revascularisation, it reduces the danger of the operation. However, in cases with large aortic annulus it must be avoided.

